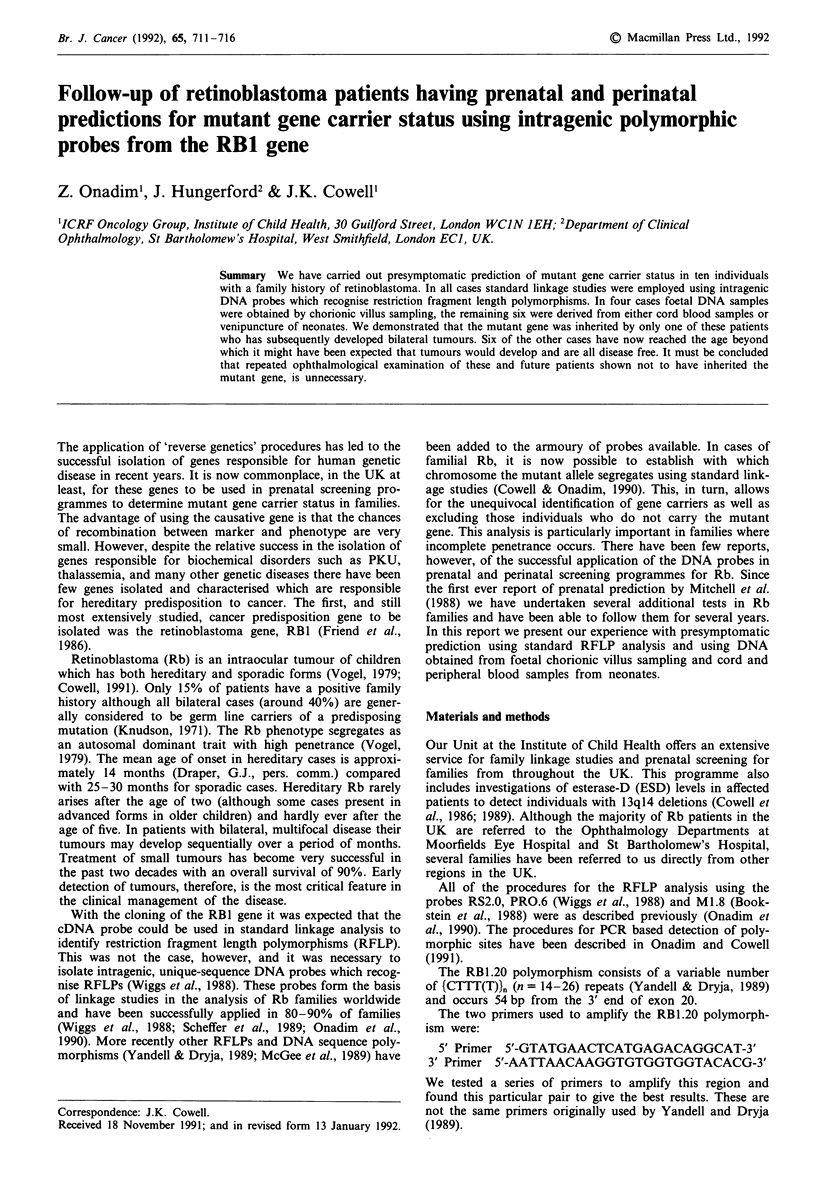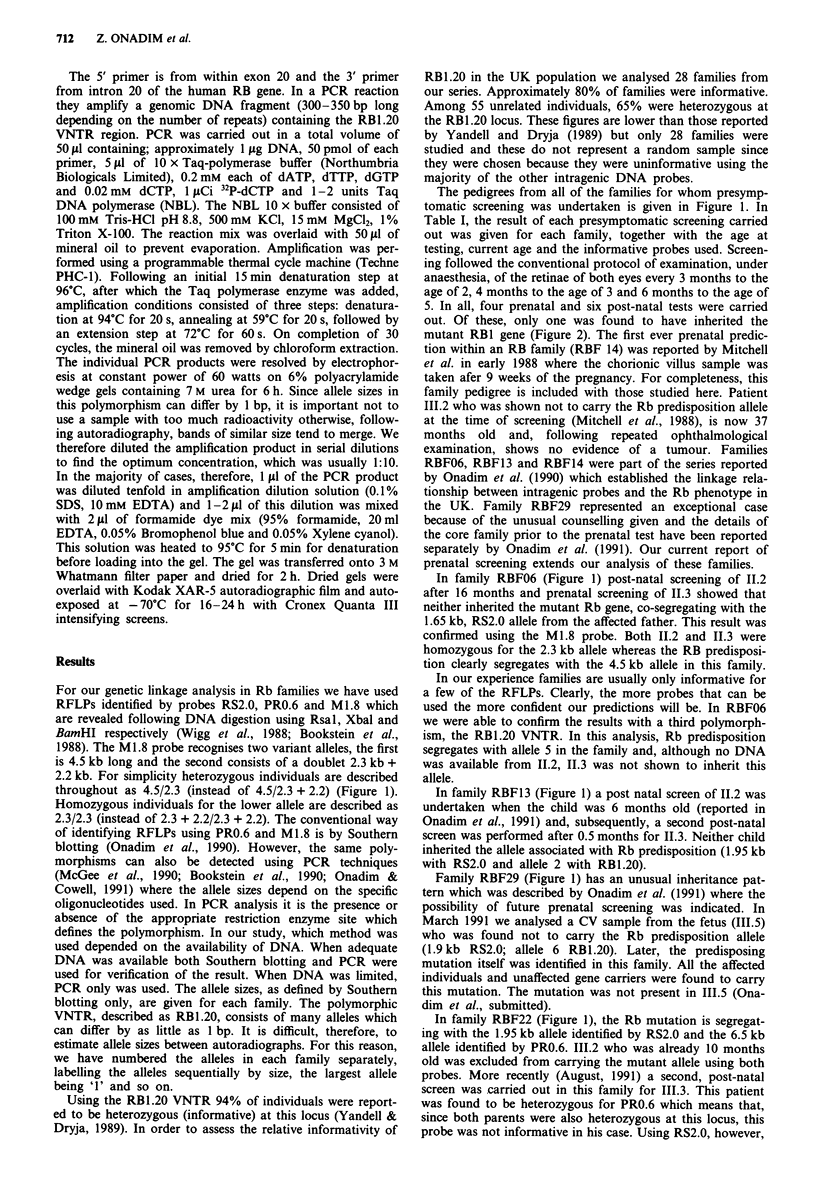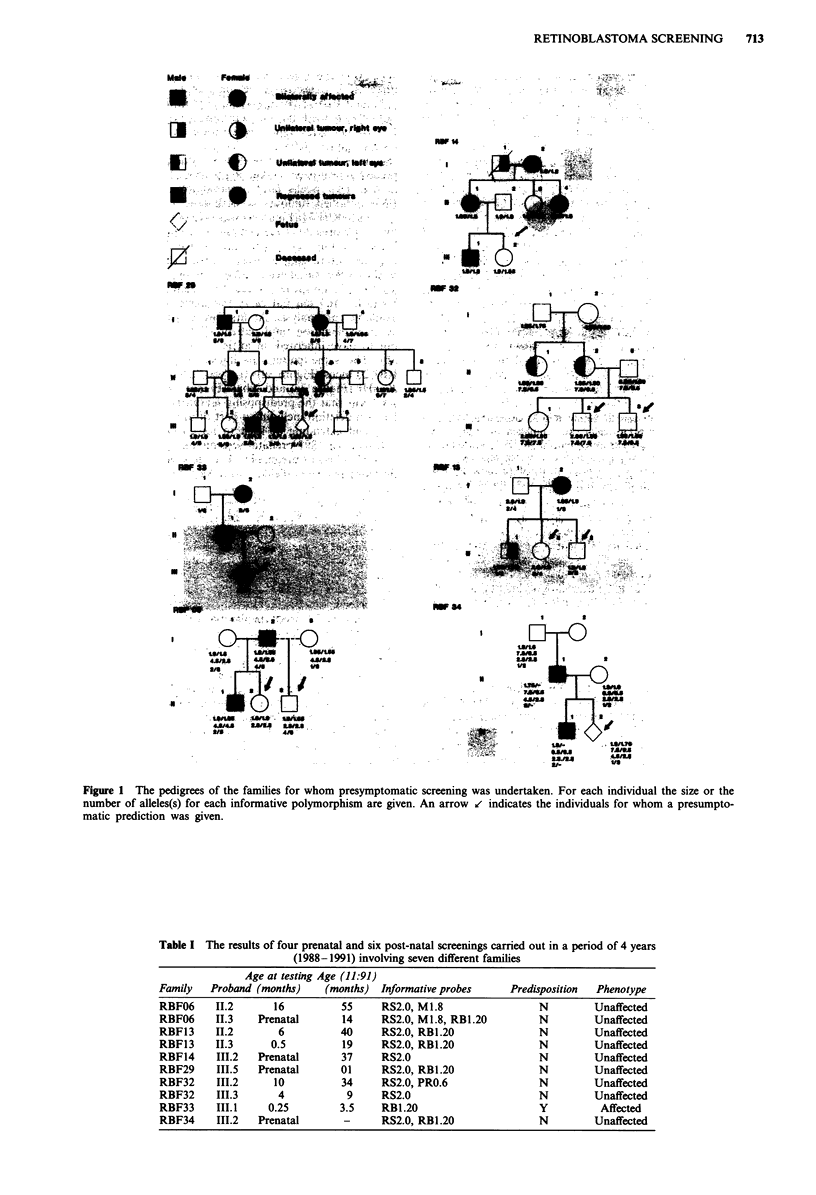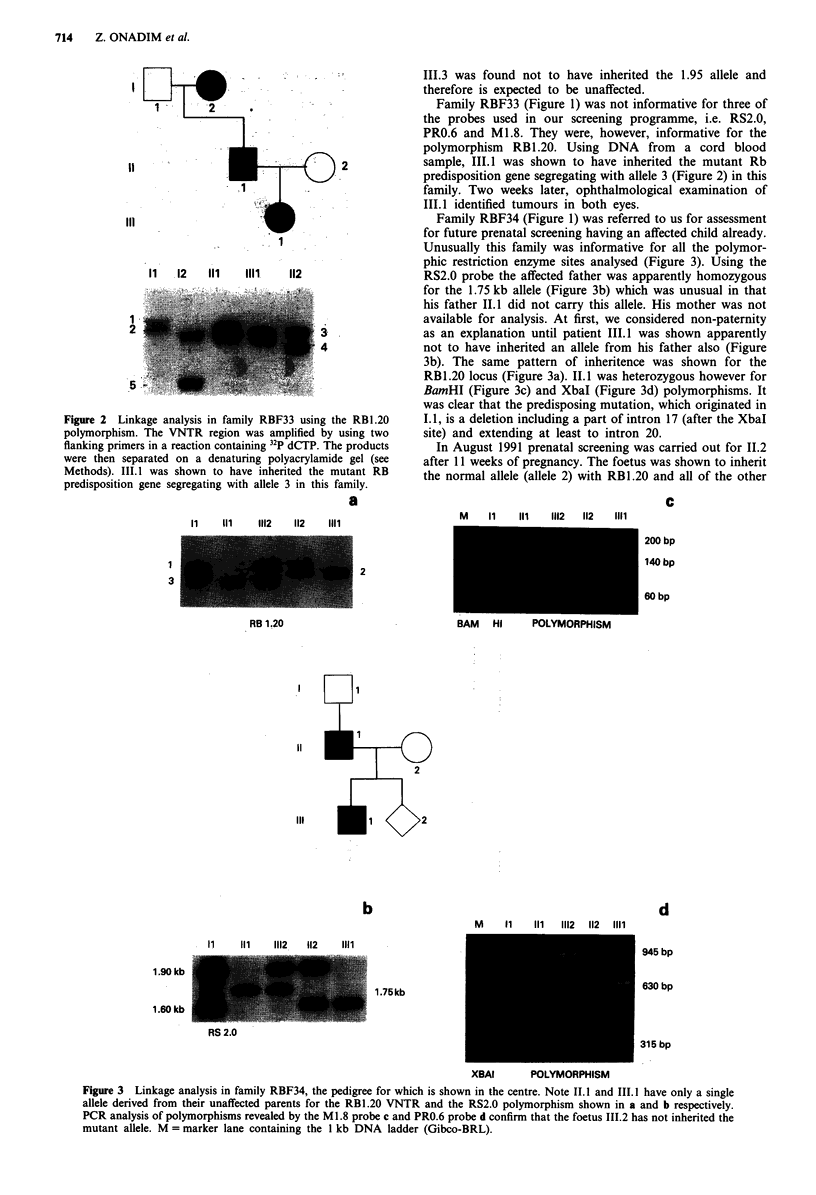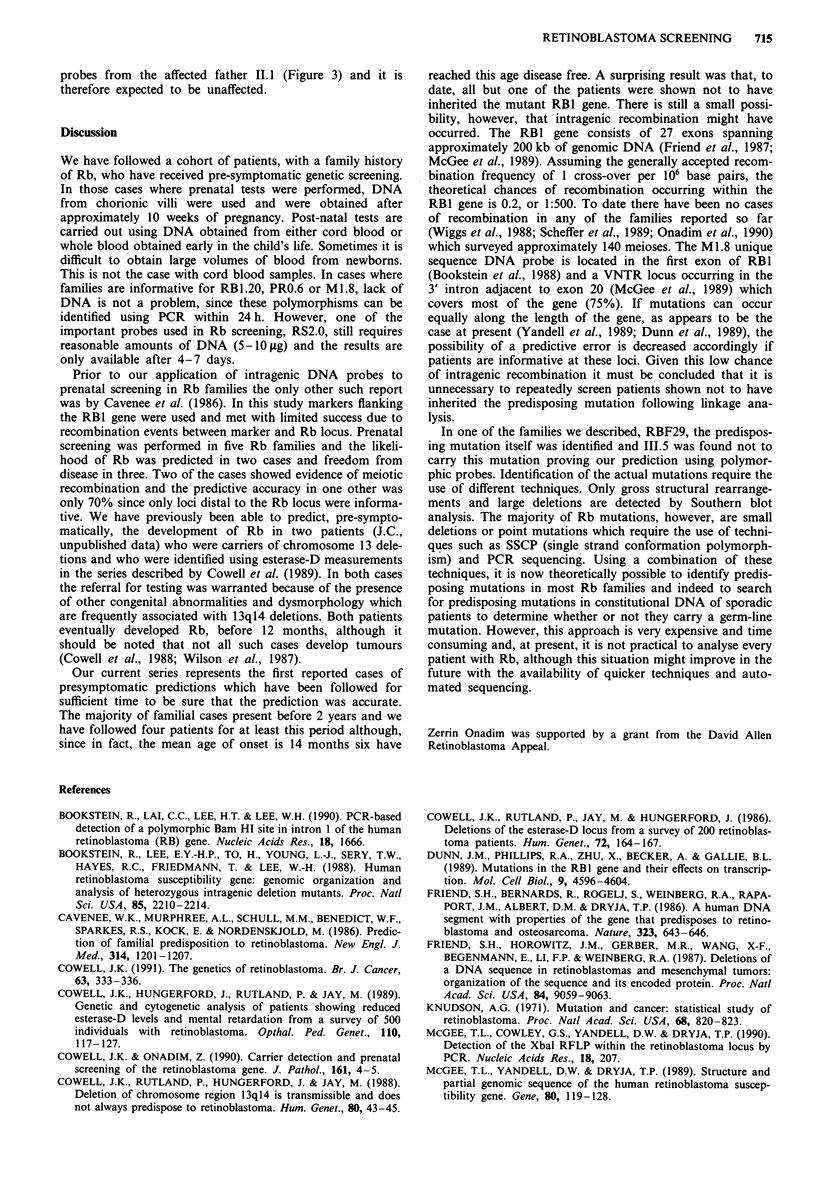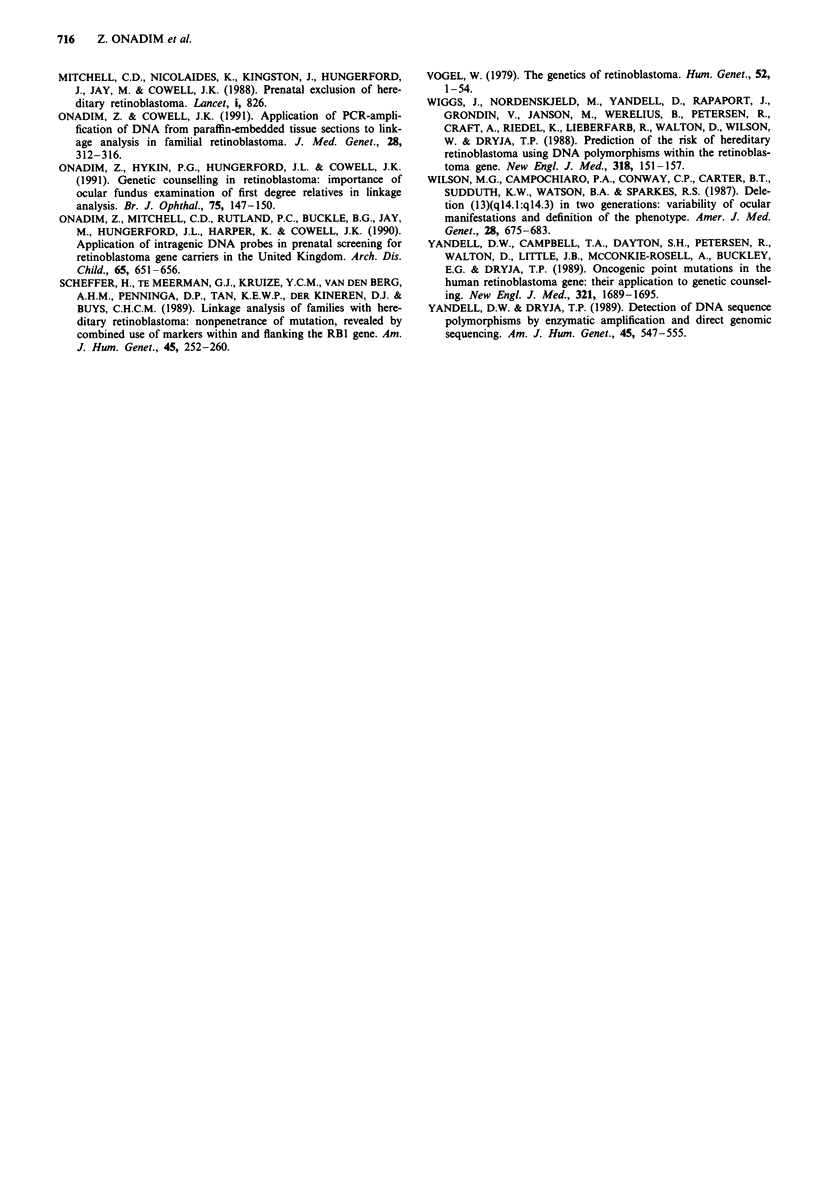# Follow-up of retinoblastoma patients having prenatal and perinatal predictions for mutant gene carrier status using intragenic polymorphic probes from the RB1 gene.

**DOI:** 10.1038/bjc.1992.150

**Published:** 1992-05

**Authors:** Z. Onadim, J. Hungerford, J. K. Cowell

**Affiliations:** ICRF Oncology Group, Institute of Child Health, London, UK.

## Abstract

**Images:**


Br. J. Cancer (1992), 65, 711–716 © Macmillan Press Ltd., 1992

### Follow-up of retinoblastoma patients having prenatal and perinatal predictions for mutant gene carrier status using intragenic polymorphic probes from the RB1 gene

Z. Onadim[1], J. Hungerford[2] & J.K. Cowell[1]

[1]*ICRF Oncology Group, Institute of Child Health, 30 Guilford Street, London WC1N 1EH;* [2]*Department of Clinical Ophthalmology, St Bartholomew's Hospital, West Smithfield, London EC1, UK.*

**Summary** We have carried out presymptomatic prediction of mutant gene carrier status in ten individuals with a family history of retinoblastoma. In all cases standard linkage studies were employed using intragenic DNA probes which recognise restriction fragment length polymorphisms. In four cases foetal DNA samples were obtained by chorionic villus sampling, the remaining six were derived from either cord blood samples or venipuncture of neonates. We demonstrated that the mutant gene was inherited by only one of these patients who has subsequently developed bilateral tumours. Six of the other cases have now reached the age beyond which it might have been expected that tumours would develop and are all disease free. It must be concluded that repeated ophthalmological examination of these and future patients shown not to have inherited the mutant gene, is unnecessary.

The application of 'reverse genetics' procedures has led to the successful isolation of genes responsible for human genetic disease in recent years. It is now commonplace, in the UK at least, for these genes to be used in prenatal screening programmes to determine mutant gene carrier status in families. The advantage of using the causative gene is that the chances of recombination between marker and phenotype are very small. However, despite the relative success in the isolation of genes responsible for biochemical disorders such as PKU, thalassemia, and many other genetic diseases there have been few genes isolated and characterised which are responsible for hereditary predisposition to cancer. The first, and still most extensively studied, cancer predisposition gene to be isolated was the retinoblastoma gene, RB1 (Friend *et al.*, 1986).

Retinoblastoma (Rb) is an intraocular tumour of children which has both hereditary and sporadic forms (Vogel, 1979; Cowell, 1991). Only 15% of patients have a positive family history although all bilateral cases (around 40%) are generally considered to be germ line carriers of a predisposing mutation (Knudson, 1971). The Rb phenotype segregates as an autosomal dominant trait with high penetrance (Vogel, 1979). The mean age of onset in hereditary cases is approximately 14 months (Draper, G.J., pers. comm.) compared with 25–30 months for sporadic cases. Hereditary Rb rarely arises after the age of two (although some cases present in advanced forms in older children) and hardly ever after the age of five. In patients with bilateral, multifocal disease their tumours may develop sequentially over a period of months. Treatment of small tumours has become very successful in the past two decades with an overall survival of 90%. Early detection of tumours, therefore, is the most critical feature in the clinical management of the disease.

With the cloning of the RB1 gene it was expected that the cDNA probe could be used in standard linkage analysis to identify restriction fragment length polymorphisms (RFLP). This was not the case, however, and it was necessary to isolate intragenic, unique-sequence DNA probes which recognise RFLPs (Wiggs *et al.*, 1988). These probes form the basis of linkage studies in the analysis of Rb families worldwide and have been successfully applied in 80–90% of families (Wiggs *et al.*, 1988; Scheffer *et al.*, 1989; Onadim *et al.*, 1990). More recently other RFLPs and DNA sequence polymorphisms (Yandell & Dryja, 1989; McGee *et al.*, 1989) have been added to the armoury of probes available. In cases of familial Rb, it is now possible to establish with which chromosome the mutant allele segregates using standard linkage studies (Cowell & Onadim, 1990). This, in turn, allows for the unequivocal identification of gene carriers as well as excluding those individuals who do not carry the mutant gene. This analysis is particularly important in families where incomplete penetrance occurs. There have been few reports, however, of the successful application of the DNA probes in prenatal and perinatal screening programmes for Rb. Since the first ever report of prenatal prediction by Mitchell *et al.* (1988) we have undertaken several additional tests in Rb families and have been able to follow them for several years. In this report we present our experience with presymptomatic prediction using standard RFLP analysis and using DNA obtained from foetal chorionic villus sampling and cord and peripheral blood samples from neonates.

Correspondence: J.K. Cowell.
Received 18 November 1991; and in revised form 13 January 1992.

### Materials and methods

Our Unit at the Institute of Child Health offers an extensive service for family linkage studies and prenatal screening for families from throughout the UK. This programme also includes investigations of esterase-D (ESD) levels in affected patients to detect individuals with 13q14 deletions (Cowell *et al.*, 1986; 1989). Although the majority of Rb patients in the UK are referred to the Ophthalmology Departments at Moorfields Eye Hospital and St Bartholomew's Hospital, several families have been referred to us directly from other regions in the UK.

All of the procedures for the RFLP analysis using the probes RS2.0, PRO.6 (Wiggs *et al.*, 1988) and M1.8 (Bookstein *et al.*, 1988) were as described previously (Onadim *et al.*, 1990). The procedures for PCR based detection of polymorphic sites have been described in Onadim and Cowell (1991).

The RB1.20 polymorphism consists of a variable number of $\{CTTT(T)\}_n$ ($n$ = 14–26) repeats (Yandell & Dryja, 1989) and occurs 54 bp from the 3′ end of exon 20.

The two primers used to amplify the RB1.20 polymorphism were:

5′ Primer 5′-GTATGAACTCATGAGACAGGCAT-3′
3′ Primer 5′-AATTAACAAGGTGTGGTGGTACACG-3′

We tested a series of primers to amplify this region and found this particular pair to give the best results. These are not the same primers originally used by Yandell and Dryja (1989).

The 5′ primer is from within exon 20 and the 3′ primer from intron 20 of the human RB gene. In a PCR reaction they amplify a genomic DNA fragment (300–350 bp long depending on the number of repeats) containing the RB1.20 VNTR region. PCR was carried out in a total volume of 50 μl containing; approximately 1 μg DNA, 50 pmol of each primer, 5 μl of 10 × Taq-polymerase buffer (Northumbria Biologicals Limited), 0.2 mM each of dATP, dTTP, dGTP and 0.02 mM dCTP, 1 μCi $^{32}$P-dCTP and 1–2 units Taq DNA polymerase (NBL). The NBL 10 × buffer consisted of 100 mM Tris-HCl pH 8.8, 500 mM KCl, 15 mM $MgCl_2$, 1% Triton X-100. The reaction mix was overlaid with 50 μl of mineral oil to prevent evaporation. Amplification was performed using a programmable thermal cycle machine (Techne PHC-1). Following an initial 15 min denaturation step at 96°C, after which the Taq polymerase enzyme was added, amplification conditions consisted of three steps: denaturation at 94°C for 20 s, annealing at 59°C for 20 s, followed by an extension step at 72°C for 60 s. On completion of 30 cycles, the mineral oil was removed by chloroform extraction. The individual PCR products were resolved by electrophoresis at constant power of 60 watts on 6% polyacrylamide wedge gels containing 7 M urea for 6 h. Since allele sizes in this polymorphism can differ by 1 bp, it is important not to use a sample with too much radioactivity otherwise, following autoradiography, bands of similar size tend to merge. We therefore diluted the amplification product in serial dilutions to find the optimum concentration, which was usually 1:10. In the majority of cases, therefore, 1 μl of the PCR product was diluted tenfold in amplification dilution solution (0.1% SDS, 10 mM EDTA) and 1–2 μl of this dilution was mixed with 2 μl of formamide dye mix (95% formamide, 20 ml EDTA, 0.05% Bromophenol blue and 0.05% Xylene cyanol). This solution was heated to 95°C for 5 min for denaturation before loading into the gel. The gel was transferred onto 3 M Whatmann filter paper and dried for 2 h. Dried gels were overlaid with Kodak XAR-5 autoradiographic film and autoexposed at −70°C for 16–24 h with Cronex Quanta III intensifying screens.

### Results

For our genetic linkage analysis in Rb families we have used RFLPs identified by probes RS2.0, PR0.6 and M1.8 which are revealed following DNA digestion using RsaI, XbaI and *Bam*HI respectively (Wigg *et al.*, 1988; Bookstein *et al.*, 1988). The M1.8 probe recognises two variant alleles, the first is 4.5 kb long and the second consists of a doublet 2.3 kb + 2.2 kb. For simplicity heterozygous individuals are described throughout as 4.5/2.3 (instead of 4.5/2.3 + 2.2) (Figure 1). Homozygous individuals for the lower allele are described as 2.3/2.3 (instead of 2.3 + 2.2/2.3 + 2.2). The conventional way of identifying RFLPs using PR0.6 and M1.8 is by Southern blotting (Onadim *et al.*, 1990). However, the same polymorphisms can also be detected using PCR techniques (McGee *et al.*, 1990; Bookstein *et al.*, 1990; Onadim & Cowell, 1991) where the allele sizes depend on the specific oligonucleotides used. In PCR analysis it is the presence or absence of the appropriate restriction enzyme site which defines the polymorphism. In our study, which method was used depended on the availability of DNA. When adequate DNA was available both Southern blotting and PCR were used for verification of the result. When DNA was limited, PCR only was used. The allele sizes, as defined by Southern blotting only, are given for each family. The polymorphic VNTR, described as RB1.20, consists of many alleles which can differ by as little as 1 bp. It is difficult, therefore, to estimate allele sizes between autoradiographs. For this reason, we have numbered the alleles in each family separately, labelling the alleles sequentially by size, the largest allele being '1' and so on.

Using the RB1.20 VNTR 94% of individuals were reported to be heterozygous (informative) at this locus (Yandell & Dryja, 1989). In order to assess the relative informativity of RB1.20 in the UK population we analysed 28 families from our series. Approximately 80% of families were informative. Among 55 unrelated individuals, 65% were heterozygous at the RB1.20 locus. These figures are lower than those reported by Yandell and Dryja (1989) but only 28 families were studied and these do not represent a random sample since they were chosen because they were uninformative using the majority of the other intragenic DNA probes.

The pedigrees from all of the families for whom presymptomatic screening was undertaken is given in Figure 1. In Table I, the result of each presymptomatic screening carried out was given for each family, together with the age at testing, current age and the informative probes used. Screening followed the conventional protocol of examination, under anaesthesia, of the retinae of both eyes every 3 months to the age of 2, 4 months to the age of 3 and 6 months to the age of 5. In all, four prenatal and six post-natal tests were carried out. Of these, only one was found to have inherited the mutant RB1 gene (Figure 2). The first ever prenatal prediction within an RB family (RBF 14) was reported by Mitchell *et al.* in early 1988 where the chorionic villus sample was taken afer 9 weeks of the pregnancy. For completeness, this family pedigree is included with those studied here. Patient III.2 who was shown not to carry the Rb predisposition allele at the time of screening (Mitchell *et al.*, 1988), is now 37 months old and, following repeated ophthalmological examination, shows no evidence of a tumour. Families RBF06, RBF13 and RBF14 were part of the series reported by Onadim *et al.* (1990) which established the linkage relationship between intragenic probes and the Rb phenotype in the UK. Family RBF29 represented an exceptional case because of the unusual counselling given and the details of the core family prior to the prenatal test have been reported separately by Onadim *et al.* (1991). Our current report of prenatal screening extends our analysis of these families.

In family RBF06 (Figure 1) post-natal screening of II.2 after 16 months and prenatal screening of II.3 showed that neither inherited the mutant Rb gene, co-segregating with the 1.65 kb, RS2.0 allele from the affected father. This result was confirmed using the M1.8 probe. Both II.2 and II.3 were homozygous for the 2.3 kb allele whereas the RB predisposition clearly segregates with the 4.5 kb allele in this family.

In our experience families are usually only informative for a few of the RFLPs. Clearly, the more probes that can be used the more confident our predictions will be. In RBF06 we were able to confirm the results with a third polymorphism, the RB1.20 VNTR. In this analysis, Rb predisposition segregates with allele 5 in the family and, although no DNA was available from II.2, II.3 was not shown to inherit this allele.

In family RBF13 (Figure 1) a post natal screen of II.2 was undertaken when the child was 6 months old (reported in Onadim *et al.*, 1991) and, subsequently, a second post-natal screen was performed after 0.5 months for II.3. Neither child inherited the allele associated with Rb predisposition (1.95 kb with RS2.0 and allele 2 with RB1.20).

Family RBF29 (Figure 1) has an unusual inheritance pattern which was described by Onadim *et al.* (1991) where the possibility of future prenatal screening was indicated. In March 1991 we analysed a CV sample from the fetus (III.5) who was found not to carry the Rb predisposition allele (1.9 kb RS2.0; allele 6 RB1.20). Later, the predisposing mutation itself was identified in this family. All the affected individuals and unaffected gene carriers were found to carry this mutation. The mutation was not present in III.5 (Onadim *et al.*, submitted).

In family RBF22 (Figure 1), the Rb mutation is segregating with the 1.95 kb allele identified by RS2.0 and the 6.5 kb allele identified by PR0.6. III.2 who was already 10 months old was excluded from carrying the mutant allele using both probes. More recently (August, 1991) a second, post-natal screen was carried out in this family for III.3. This patient was found to be heterozygous for PR0.6 which means that, since both parents were also heterozygous at this locus, this probe was not informative in his case. Using RS2.0, however,

**Figure 1** The pedigrees of the families for whom presymptomatic screening was undertaken. For each individual the size or the number of alleles(s) for each informative polymorphism are given. An arrow ↙ indicates the individuals for whom a presumptomatic prediction was given.

**Table I** The results of four prenatal and six post-natal screenings carried out in a period of 4 years (1988–1991) involving seven different families

| *Family* | *Proband* | *Age at testing (months)* | *Age (11:91) (months)* | *Informative probes* | *Predisposition* | *Phenotype* |
|---|---|---|---|---|---|---|
| RBF06 | II.2 | 16 | 55 | RS2.0, M1.8 | N | Unaffected |
| RBF06 | II.3 | Prenatal | 14 | RS2.0, M1.8, RB1.20 | N | Unaffected |
| RBF13 | II.2 | 6 | 40 | RS2.0, RB1.20 | N | Unaffected |
| RBF13 | II.3 | 0.5 | 19 | RS2.0, RB1.20 | N | Unaffected |
| RBF14 | III.2 | Prenatal | 37 | RS2.0 | N | Unaffected |
| RBF29 | III.5 | Prenatal | 01 | RS2.0, RB1.20 | N | Unaffected |
| RBF32 | III.2 | 10 | 34 | RS2.0, PR0.6 | N | Unaffected |
| RBF32 | III.3 | 4 | 9 | RS2.0 | N | Unaffected |
| RBF33 | III.1 | 0.25 | 3.5 | RB1.20 | Y | Affected |
| RBF34 | III.2 | Prenatal | – | RS2.0, RB1.20 | N | Unaffected |

**Figure 2** Linkage analysis in family RBF33 using the RB1.20 polymorphism. The VNTR region was amplified by using two flanking primers in a reaction containing $^{32}P$ dCTP. The products were then separated on a denaturing polyacrylamide gel (see Methods). III.1 was shown to have inherited the mutant RB predisposition gene segregating with allele 3 in this family.

III.3 was found not to have inherited the 1.95 allele and therefore is expected to be unaffected.

Family RBF33 (Figure 1) was not informative for three of the probes used in our screening programme, i.e. RS2.0, PR0.6 and M1.8. They were, however, informative for the polymorphism RB1.20. Using DNA from a cord blood sample, III.1 was shown to have inherited the mutant Rb predisposition gene segregating with allele 3 (Figure 2) in this family. Two weeks later, ophthalmological examination of III.1 identified tumours in both eyes.

Family RBF34 (Figure 1) was referred to us for assessment for future prenatal screening having an affected child already. Unusually this family was informative for all the polymorphic restriction enzyme sites analysed (Figure 3). Using the RS2.0 probe the affected father was apparently homozygous for the 1.75 kb allele (Figure 3b) which was unusual in that his father II.1 did not carry this allele. His mother was not available for analysis. At first, we considered non-paternity as an explanation until patient III.1 was shown apparently not to have inherited an allele from his father also (Figure 3b). The same pattern of inheritence was shown for the RB1.20 locus (Figure 3a). II.1 was heterozygous however for *Bam*HI (Figure 3c) and XbaI (Figure 3d) polymorphisms. It was clear that the predisposing mutation, which originated in I.1, is a deletion including a part of intron 17 (after the XbaI site) and extending at least to intron 20.

In August 1991 prenatal screening was carried out for II.2 after 11 weeks of pregnancy. The foetus was shown to inherit the normal allele (allele 2) with RB1.20 and all of the other

**Figure 3** Linkage analysis in family RBF34, the pedigree for which is shown in the centre. Note II.1 and III.1 have only a single allele derived from their unaffected parents for the RB1.20 VNTR and the RS2.0 polymorphism shown in **a** and **b** respectively. PCR analysis of polymorphisms revealed by the M1.8 probe **c** and PR0.6 probe **d** confirm that the foetus III.2 has not inherited the mutant allele. M = marker lane containing the 1 kb DNA ladder (Gibco-BRL).

probes from the affected father II.1 (Figure 3) and it is therefore expected to be unaffected.

### Discussion

We have followed a cohort of patients, with a family history of Rb, who have received pre-symptomatic genetic screening. In those cases where prenatal tests were performed, DNA from chorionic villi were used and were obtained after approximately 10 weeks of pregnancy. Post-natal tests are carried out using DNA obtained from either cord blood or whole blood obtained early in the child's life. Sometimes it is difficult to obtain large volumes of blood from newborns. This is not the case with cord blood samples. In cases where families are informative for RB1.20, PR0.6 or M1.8, lack of DNA is not a problem, since these polymorphisms can be identified using PCR within 24 h. However, one of the important probes used in Rb screening, RS2.0, still requires reasonable amounts of DNA (5–10 μg) and the results are only available after 4–7 days.

Prior to our application of intragenic DNA probes to prenatal screening in Rb families the only other such report was by Cavenee *et al.* (1986). In this study markers flanking the RB1 gene were used and met with limited success due to recombination events between marker and Rb locus. Prenatal screening was performed in five Rb families and the likelihood of Rb was predicted in two cases and freedom from disease in three. Two of the cases showed evidence of meiotic recombination and the predictive accuracy in one other was only 70% since only loci distal to the Rb locus were informative. We have previously been able to predict, pre-symptomatically, the development of Rb in two patients (J.C., unpublished data) who were carriers of chromosome 13 deletions and who were identified using esterase-D measurements in the series described by Cowell *et al.* (1989). In both cases the referral for testing was warranted because of the presence of other congenital abnormalities and dysmorphology which are frequently associated with 13q14 deletions. Both patients eventually developed Rb, before 12 months, although it should be noted that not all such cases develop tumours (Cowell *et al.*, 1988; Wilson *et al.*, 1987).

Our current series represents the first reported cases of presymptomatic predictions which have been followed for sufficient time to be sure that the prediction was accurate. The majority of familial cases present before 2 years and we have followed four patients for at least this period although, since in fact, the mean age of onset is 14 months six have reached this age disease free. A surprising result was that, to date, all but one of the patients were shown not to have inherited the mutant RB1 gene. There is still a small possibility, however, that intragenic recombination might have occurred. The RB1 gene consists of 27 exons spanning approximately 200 kb of genomic DNA (Friend *et al.*, 1987; McGee *et al.*, 1989). Assuming the generally accepted recombination frequency of 1 cross-over per $10^6$ base pairs, the theoretical chances of recombination occurring within the RB1 gene is 0.2, or 1:500. To date there have been no cases of recombination in any of the families reported so far (Wiggs *et al.*, 1988; Scheffer *et al.*, 1989; Onadim *et al.*, 1990) which surveyed approximately 140 meioses. The M1.8 unique sequence DNA probe is located in the first exon of RB1 (Bookstein *et al.*, 1988) and a VNTR locus occurring in the 3′ intron adjacent to exon 20 (McGee *et al.*, 1989) which covers most of the gene (75%). If mutations can occur equally along the length of the gene, as appears to be the case at present (Yandell *et al.*, 1989; Dunn *et al.*, 1989), the possibility of a predictive error is decreased accordingly if patients are informative at these loci. Given this low chance of intragenic recombination it must be concluded that it is unnecessary to repeatedly screen patients shown not to have inherited the predisposing mutation following linkage analysis.

In one of the families we described, RBF29, the predisposing mutation itself was identified and III.5 was found not to carry this mutation proving our prediction using polymorphic probes. Identification of the actual mutations require the use of different techniques. Only gross structural rearrangements and large deletions are detected by Southern blot analysis. The majority of Rb mutations, however, are small deletions or point mutations which require the use of techniques such as SSCP (single strand conformation polymorphism) and PCR sequencing. Using a combination of these techniques, it is now theoretically possible to identify predisposing mutations in most Rb families and indeed to search for predisposing mutations in constitutional DNA of sporadic patients to determine whether or not they carry a germ-line mutation. However, this approach is very expensive and time consuming and, at present, it is not practical to analyse every patient with Rb, although this situation might improve in the future with the availability of quicker techniques and automated sequencing.

Zerrin Onadim was supported by a grant from the David Allen Retinoblastoma Appeal.

### References

BOOKSTEIN, R., LAI, C.C., LEE, H.T. & LEE, W.H. (1990). PCR-based detection of a polymorphic Bam HI site in intron 1 of the human retinoblastoma (RB) gene. *Nucleic Acids Res.*, **18**, 1666.

BOOKSTEIN, R., LEE, E.Y.-H.P., TO, H., YOUNG, L.-J., SERY, T.W., HAYES, R.C., FRIEDMANN, T. & LEE, W.-H. (1988). Human retinoblastoma susceptibility gene: genomic organization and analysis of heterozygous intragenic deletion mutants. *Proc. Natl Sci. USA*, **85**, 2210–2214.

CAVENEE, W.K., MURPHREE, A.L., SCHULL, M.M., BENEDICT, W.F., SPARKES, R.S., KOCK, E. & NORDENSKJOLD, M. (1986). Prediction of familial predisposition to retinoblastoma. *New Engl. J. Med.*, **314**, 1201–1207.

COWELL, J.K. (1991). The genetics of retinoblastoma. *Br. J. Cancer*, **63**, 333–336.

COWELL, J.K., HUNGERFORD, J., RUTLAND, P. & JAY, M. (1989). Genetic and cytogenetic analysis of patients showing reduced esterase-D levels and mental retardation from a survey of 500 individuals with retinoblastoma. *Opthal. Ped. Genet.*, **110**, 117–127.

COWELL, J.K. & ONADIM, Z. (1990). Carrier detection and prenatal screening of the retinoblastoma gene. *J. Pathol.*, **161**, 4–5.

COWELL, J.K., RUTLAND, P., HUNGERFORD, J. & JAY, M. (1988). Deletion of chromosome region 13q14 is transmissible and does not always predispose to retinoblastoma. *Hum. Genet.*, **80**, 43–45.

COWELL, J.K., RUTLAND, P., JAY, M. & HUNGERFORD, J. (1986). Deletions of the esterase-D locus from a survey of 200 retinoblastoma patients. *Hum. Genet.*, **72**, 164–167.

DUNN, J.M., PHILLIPS, R.A., ZHU, X., BECKER, A. & GALLIE, B.L. (1989). Mutations in the RB1 gene and their effects on transcription. *Mol. Cell Biol.*, **9**, 4596–4604.

FRIEND, S.H., BERNARDS, R., ROGELJ, S., WEINBERG, R.A., RAPAPORT, J.M., ALBERT, D.M. & DRYJA, T.P. (1986). A human DNA segment with properties of the gene that predisposes to retinoblastoma and osteosarcoma. *Nature*, **323**, 643–646.

FRIEND, S.H., HOROWITZ, J.M., GERBER, M.R., WANG, X-F., BEGENMANN, E., LI, F.P. & WEINBERG, R.A. (1987). Deletions of a DNA sequence in retinoblastomas and mesenchymal tumors: organization of the sequence and its encoded protein. *Proc. Natl Acad. Sci. USA*, **84**, 9059–9063.

KNUDSON, A.G. (1971). Mutation and cancer: statistical study of retinoblastoma. *Proc. Natl Acad. Sci. USA*, **68**, 820–823.

MCGEE, T.L., COWLEY, G.S., YANDELL, D.W. & DRYJA, T.P. (1990). Detection of the XbaI RFLP within the retinoblastoma locus by PCR. *Nucleic Acids Res.*, **18**, 207.

MCGEE, T.L., YANDELL, D.W. & DRYJA, T.P. (1989). Structure and partial genomic sequence of the human retinoblastoma susceptibility gene. *Gene*, **80**, 119–128.

MITCHELL, C.D., NICOLAIDES, K., KINGSTON, J., HUNGERFORD, J., JAY, M. & COWELL, J.K. (1988). Prenatal exclusion of hereditary retinoblastoma. *Lancet*, **i**, 826.

ONADIM, Z. & COWELL, J.K. (1991). Application of PCR-amplification of DNA from paraffin-embedded tissue sections to linkage analysis in familial retinoblastoma. *J. Med. Genet.*, **28**, 312–316.

ONADIM, Z., HYKIN, P.G., HUNGERFORD, J.L. & COWELL, J.K. (1991). Genetic counselling in retinoblastoma: importance of ocular fundus examination of first degree relatives in linkage analysis. *Br. J. Ophthal.*, **75**, 147–150.

ONADIM, Z., MITCHELL, C.D., RUTLAND, P.C., BUCKLE, B.G., JAY, M., HUNGERFORD, J.L., HARPER, K. & COWELL, J.K. (1990). Application of intragenic DNA probes in prenatal screening for retinoblastoma gene carriers in the United Kingdom. *Arch. Dis. Child.*, **65**, 651–656.

SCHEFFER, H., TE MEERMAN, G.J., KRUIZE, Y.C.M., VAN DEN BERG, A.H.M., PENNINGA, D.P., TAN, K.E.W.P., DER KINEREN, D.J. & BUYS, C.H.C.M. (1989). Linkage analysis of families with hereditary retinoblastoma: nonpenetrance of mutation, revealed by combined use of markers within and flanking the RB1 gene. *Am. J. Hum. Genet.*, **45**, 252–260.

VOGEL, W. (1979). The genetics of retinoblastoma. *Hum. Genet.*, **52**, 1–54.

WIGGS, J., NORDENSKJELD, M., YANDELL, D., RAPAPORT, J., GRONDIN, V., JANSON, M., WERELIUS, B., PETERSEN, R., CRAFT, A., RIEDEL, K., LIEBERFARB, R., WALTON, D., WILSON, W. & DRYJA, T.P. (1988). Prediction of the risk of hereditary retinoblastoma using DNA polymorphisms within the retinoblastoma gene. *New Engl. J. Med.*, **318**, 151–157.

WILSON, M.G., CAMPOCHIARO, P.A., CONWAY, C.P., CARTER, B.T., SUDDUTH, K.W., WATSON, B.A. & SPARKES, R.S. (1987). Deletion (13)(q14.1:q14.3) in two generations: variability of ocular manifestations and definition of the phenotype. *Amer. J. Med. Genet.*, **28**, 675–683.

YANDELL, D.W., CAMPBELL, T.A., DAYTON, S.H., PETERSEN, R., WALTON, D., LITTLE, J.B., MCCONKIE-ROSELL, A., BUCKLEY, E.G. & DRYJA, T.P. (1989). Oncogenic point mutations in the human retinoblastoma gene: their application to genetic counseling. *New Engl. J. Med.*, **321**, 1689–1695.

YANDELL, D.W. & DRYJA, T.P. (1989). Detection of DNA sequence polymorphisms by enzymatic amplification and direct genomic sequencing. *Am. J. Hum. Genet.*, **45**, 547–555.